# Small-angle X-ray scattering characteristics of mouse brain: Planar imaging measurements and tomographic imaging simulations

**DOI:** 10.1371/journal.pone.0186451

**Published:** 2017-10-31

**Authors:** Mina Choi, Bahaa Ghammraoui, Aldo Badano

**Affiliations:** 1 Fischell Department of Bioengineering, University of Maryland, College Park, MD, 20742, United States of America; 2 Division of Imaging, Diagnostics, and Software Reliability, Office of Science and Engineering Laboratories, CDRH/USFDA, Silver Spring, Maryland 20993, United States of America; Massey University, NEW ZEALAND

## Abstract

Small-angle x-ray scattering (SAXS) imaging can differentiate tissue types based on their nanoscale molecular structure. However, characterization of the coherent scattering cross-section profile of relevant tissues is needed to optimally design SAXS imaging techniques for a variety of biomedical applications. Reported measured nervous tissue x-ray scattering cross sections under a synchrotron source have had limited agreement. We report a set of x-ray cross-section measurements obtained from planar SAXS imaging of 1 mm thick mouse brain (APP/PS1 wild-type) coronal slices using an 8 keV laboratory x-ray source. Two characteristic peaks were found at 0.96 and 1.60 nm^−1^ attributed to myelin. The peak intensities varied by location in the slice. We found that regions of gray matter, white matter, and corpus callosum could be segmented by their increasing intensities of myelin peaks respectively. Measured small-angle x-ray scattering cross sections were then used to define brain tissue scattering properties in a GPU-accelerated Monte Carlo simulation of SAXS computed tomography (CT) using a higher monochromatic x-ray energy (20 keV) to study design trade-offs for noninvasive *in vivo* SAXS imaging on a small-animal head including radiation dose, signal-to-noise ratio (SNR), and the effect of skull presence on the previous two metrics. Simulation results show the estimated total dose to the mouse head for a single SAXS-CT slice was 149.4 mGy. The pixel SNR was approximately 30.8 for white matter material whether or not a skull was present. In this early-stage proof-of-principle work, we have demonstrated our brain cross-section data and simulation tools can be used to assess optimal instrument parameters for dedicated small-animal SAXS-CT prototypes.

## Introduction

Reliable characterization and imaging of brain tissue structure is key to the understanding and cure of neurodegenerative diseases. [1, 2] Small-angle x-ray scattering (SAXS) techniques measure coherently scattered x-ray deflections at small angles analyzed to produce nanometer-scale structural information (0.1-100 nm) about the scattering sample. [3] Recently, efforts have been focused on utilizing SAXS for medical imaging to provide better material characterization and for diagnostic applications. Since x rays carry higher energies than visible light, SAXS imaging has potential to non-invasively image deeper tissues beyond a millimeter.

Conventional x-ray medical imaging techniques have primarily focused on differentiating materials based on absorption properties providing micrometer scale morphology. However, absorption-based imaging approaches are limited in that pathology often shares similar attenuation characteristics with normal surrounding anatomy, especially during early disease stages where change occurs at the molecular and cellular levels. There is increasing interest in measuring and utilizing scattered x rays, traditionally considered noise in absorption-based approaches, for nanometer-scale structural information coupled with micrometer scale spatial information with the ultimate goal of improving image quality and diagnostic performance.

**Fig 1:**
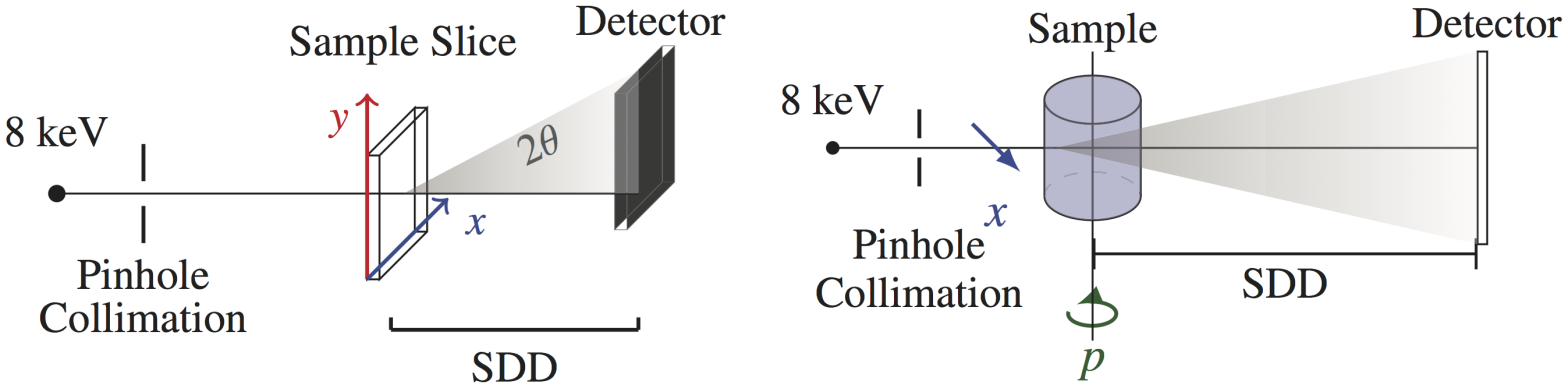
Schematic of the imaging setup for planar SAXS (left) and SAXS-CT (right).

In transmission SAXS, an x-ray pencil beam traverses a sample and scattering patterns are recorded at small angles on a 1D or 2D detector. As shown in Fig 1(*left*) Planar SAXS (pSAXS) might use stepper motors to position and collect SAXS data at various locations in the plane orthogonal to the beam direction. This information could be used to map and differentiate materials by their inherent scattering cross section. Several research groups have investigated this approach for studying nanostructure characterization of bone, [4, 5] and cardiac tissue [6], and rat brain slices. [7] However SAXS signal quality and resolution are affected by sample thickness and therefore applications of pSAXS have been limited to *ex vivo* biopsy studies. To contribute to the improvement of this new technique, we recently reported on imaging phantoms for the assessment of pSAXS image quality. [8]

A different approach is depicted in Fig 1(*right*). Here, a SAXS computed tomography (SAXS-CT) design uses image reconstruction algorithms to obtain SAXS profiles of locations deep within objects enabling applications in *in vivo* molecular x-ray imaging. This technique has been used to study biological tissues and plastics, [9] polyethelene, [10] collagen-based phantoms, [11] lamb tissue, [12] and rat brain tissue. [13, 14] We have recently explored a method to assess SAXS data quality using Monte Carlo imaging simulations. [15]

An area of interest in clinical applications of SAXS-CT is the study and diagnosis of neurological disorders. There are currently no known cures or effective treatment for many neurological disorders. Recent discoveries indicate that biomolecular changes may appear 20 or more years before dementia symptoms appear. [1] In this context, we propose SAXS imaging may be able to detect earlier disease changes and assist in the study of therapy effectiveness. [16] The most notable and pronounced potential biomarker is myelin, a highly structured fibrous tissue that has been investigated using SAXS for multiple sclerosis. [13, 17, 18] In addition, amyloid fibers [19, 20] have been investigated for imaging Alzheimer’s disease along with SAXS signals of brain tumours. [21] To assist in these efforts, we have discovered and developed an amyloid target surrogate consisting of a much more available bovine serum albumin for utilization in phantom designs for diagnostic applications of Alzheimer’s disease. [22]

Other brain imaging methods include optical techniques that can successfully characterize molecular neurological hallmarks but lack the ability to image deep tissue where the hallmarks tend to form during early stages of disease. On the other hand, PET imaging has become the standard of practice for *in vivo* imaging using amyloid-targeting tracers. However, PET suffers from inherently low spatial resolution and low specificity. [23] MRI techniques, on the other hand, have high spatial resolution (up to micron resolution). MRI is currently utilized to study myelin density and location, [24, 25] but is not yet able to characterize nanoscale structural information.

The major setback to SAXS-CT imaging translation is the long measurement times as each position measured could take minutes to hours to obtain sufficient scatter signal. Increasing the number of scan steps improves the image resolution and increasing the number of projections improves the quality of the CT reconstruction, however, this would have a multiplicative effect on measurement times. The recent advent of 2D x-ray spectroscopic detectors that removes the necessity for x-ray monochromation and allows for more efficient use of x rays produced by implementing a hybrid angular-dispersive, energy-dispersive mode. This technology and approach may significantly reduce measurement times to make SAXS-CT practically feasible for *in vivo* applications.

In this article, we image mouse brain coronal slices using pSAXS on a laboratory source and report cross sections differences in different spatial regions of the area imaged. Measured cross sections of gray matter, white matter, and mouse skull are utilized in Monte Carlo x-ray transport simulations of SAXS-CT to explore feasibility of *in vivo* methods by estimating radiation dose and signal-to-noise ratio (SNR) of the produced images.

## Materials and methods

### 0.1 Measurement technique

We use a laboratory SAXS system (SAXSpace, Anton Paar, Ashland, VA, USA) for pSAXS measurements (Fig 1, *left*). The instrument utilizes a sealed Cu-anode tube optimized for K_*α*_ radiation (λ = 0.154 nm). The system was configured in point collimation mode with an accessible *q* range of 0.01–20 nm^−1^ (*q* = 4*πsin*(*θ*)/λ). A pinhole aperture was achieved using blocks to approximately 200×200 *μ*m. We utilized 3 stepper motors with 25 *μ*m step resolution within the instrument vacuum sample chamber to control horizontal and vertical sample motion with respect to a stationary x-ray beam. Each position was measured for 300 s. The imaging detector is a CCD camera with a pixel pitch of 24 *μ*m in an array of 2084×2084 pixels coupled with a Gd_2_O_2_S:Tb phosphor screen designed for 8-keV x rays.

We scanned two 1 mm thick, coronal slices of wild-type mouse brain placed in a tissue sample holder (Anton Paar, Ashland, VA, USA). The sample holder has x-ray transparent windows and allows the tissue to remain at atmospheric pressure while in the beam path. The mice brains were prepared by fixing in paraformaldehyde, slicing using a vibratome to 1 mm, and was stored in a phosphate buffered solution at 4°C until measurements. No staining was performed to this tissue. We measured a wide q-range of 0.53–18.3 nm^−1^ with 8x8 binning of the detector for the first slice, and a smaller q-range of 0.13–7.09 nm^−1^ with 4x4 binning for the second slice. Each slice took approximately 3-4 days to measure. The anatomy of brain slices were estimated by associating structures in the photograph images to an available Allen Developing Mouse Brain Atlas. [26]

Because registration of a particular brain tissue type is difficult once inside the SAXS system, it was necessary to image the brain slice in a 2D scanning SAXS, so we could register a particular cross-section measurement to a location in the brain. Photographs were taken of the brain slice before measurements. The tissue dehydrated over a few hours outside a buffer solution. We found that after a slice of brain dries, it could be re-hydrated by storing in phosphate buffered solution for a few hours. However to prevent temporal effects from the drying process affecting measurements, we waited until the tissue was fully dehydrated before starting measurements.

**Fig 2:**
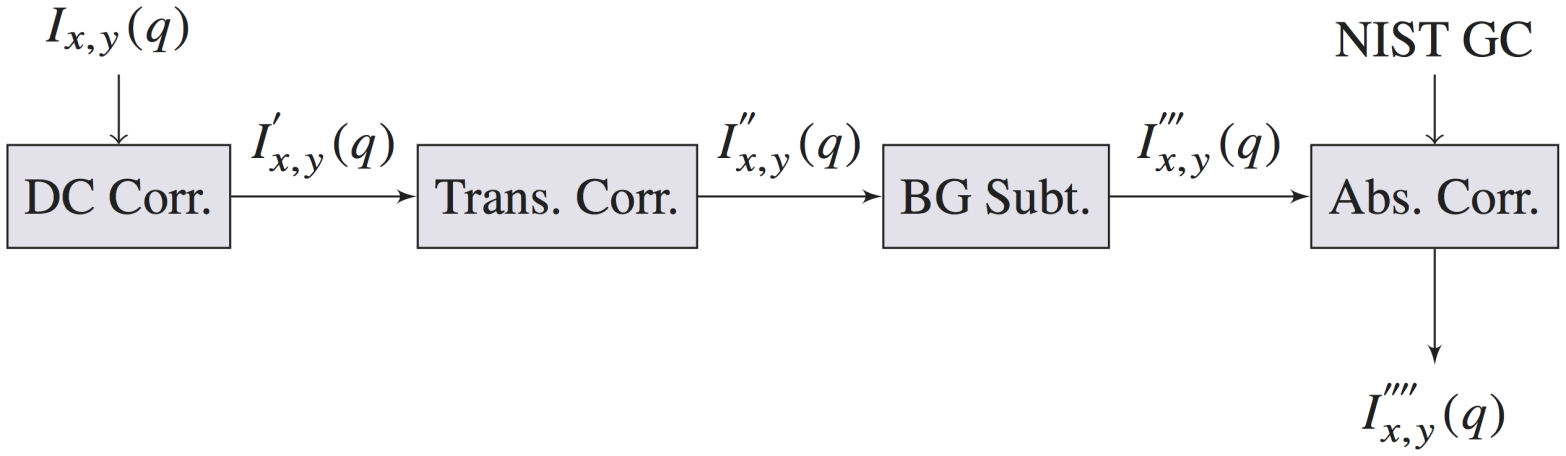
Block diagram of pSAXS data processing.

A beamstop was positioned 5 cm in front of the detector to attenuate a portion of the primary beam of transmitted x rays preventing saturation of the detector pixels. All portions of the beam path were enclosed in a vacuum-sealed chamber at below 34 mbar. The acquired 2D image of the scattering was corrected to account for standard geometric corrections due to instrument geometry and the CCD using SAXStreat (Anton Paar, Ashland, VA, USA). Then, the data was radially averaged and reduced to 1D scatter profiles, *I*(*q*). We performed four additional important corrections for our data at each coordinate pixel position (*x*, *y*) as shown in Fig 2 and are described in the following section.

### 0.2 Data processing

**Fig 3:**
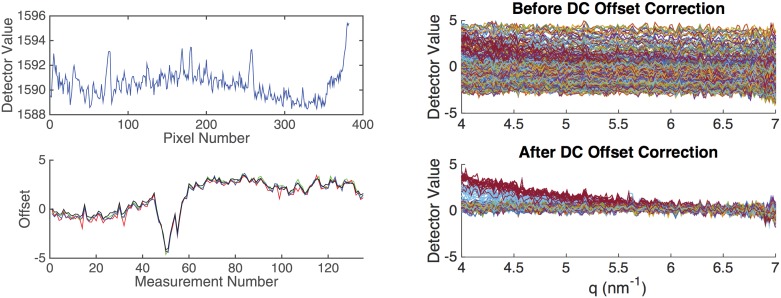
(Top left) Dark current (DC) measurement. (Bottom left) Plot of detector values after dark current subtraction at a few angular positions for all scatter measurements (6.52, 6.54, 6.56 *nm*^−1^). The black line is an average of 31 angular positions ranging from 6.52 to 7.08 *nm*^−1^. (Top right) All measurements plotted by *q* after dark current subtraction, but before dark current offset correction. (Bottom right) All measurements plotted by *q* after dark current subtraction, and after dark current offset correction.

For each set of measurements, a dark current measurement was acquired for the same exposure as each position. Ideally, a dark current measurement would be obtained immediately after each measurement to have the most accurate dark current correction due to temporal effects. However, due to the large number of subsequent measurements required per scan, it is impractical to measure a dark current between each measurement. Because the shape of the dark current 1D curve does not change other than a temperature and time-dependent offset, a reasonable compromise is to record a dark current measurement with the same exposure time at the end of each set of scanning measurements. Dark current shift over the scan time was accounted for by selecting an angular position with no signal (0 m^−1^ s^−1^) and subtracting a time-dependent offset to bring that intensity to 0 m^−1^ s^−1^. Fig 3 shows our dark current signal and the detector value at a *q* of 6 nm^−1^ for each SAXS measurement in a typical set of scans. The following equation shows the subtraction of the dark current signal and an offset,

$$\begin{array}{c} I_{x,y}^{{}^{\prime }}=I_{x,y}\left(q\right)-D_{c}\left(q\right)-{\text{offset}}_{x,y}. \end{array}$$ (1)

Each position had the same exposure time, but there were slight variations in thickness in the slice especially after drying. We corrected for thickness differences in the tissue by dividing scatter profiles by the transmission value for each pixel,

$$\begin{array}{c} I_{x,y}^{{}^{\prime \prime }}\left(q\right)=\frac{I_{x,y}^{{}^{\prime }}\left(q\right)}{I_{x,y}^{{}^{\prime }}\left(0\right)}. \end{array}$$ (2)

However, we make the assumption is that the tissue at each location has approximately the same attenuation properties.

When scatter profiles at each location are corrected for dark current and transmission differences, the background signal can be subtracted. We define the background as the windows of the tissue sample holder that contribute to the scatter signal measured. In the planar scan, we ensured that there are locations measured that only contain the windows and no tissue. We averaged the scatter profile of all pixels that only contained window, *I*_*BG*_, and subtracted this background signal from all positions,

$$\begin{array}{c} I_{x,y}^{{}^{\prime \prime \prime }}\left(q\right)=I_{x,y}^{{}^{\prime \prime }}\left(q\right)-I_{BG}^{{}^{\prime \prime }}\left(q\right). \end{array}$$ (3)

To convert empirical measurements to absolute cross sections, measurements of a secondary intensity standard, 1 mm thick glassy carbon, [27] and *q* calibration reference [28], silver behenate (AgBe), were also acquired in the same scan. The glassy carbon measurements were scaled to NIST data of absolute glassy carbon values and a calibration factor, *C*_*f*_ was obtained. The calibration factor was multiplied by all other measurements,

$$\begin{array}{c} I_{x,y}^{{}^{⁗}}\left(q\right)=C_{f}I_{x,y}^{{}^{\prime \prime \prime }}\left(q\right). \end{array}$$ (4)

The *q* angles were corrected by the AgBe measurements where peak locations are known.

### 0.3 Monte Carlo simulations of SAXS-CT imaging

To study radiation dose and signal-to-noise ratio of SAXS-CT images produced for brain imaging applications, simulations of x-ray transport of the entire SAXS imaging chain were performed using Monte Carlo techniques. We used MC-GPU, a GPU-accelerated x-ray transport simulation tool that has previously been used to generate clinically-realistic projection images and computed tomography (CT) scans of the human anatomy. [29] The code is publicly available and distributed for free in source form. MC-GPU massively multithreads a Monte Carlo simulation algorithm for the transport of x rays in a voxelized geometry utilizing x-ray interaction models and cross sections from PENELOPE 2006. [30] MC-GPU has handled realistic human anatomy phantoms, like the freely available Virtual Family model, [31] and adapted to simulate coherent scattering CT incorporating molecular form factor and structure factor effects. [15, 32, 33] We used an infinitely small monochromatic pinhole beam which was set to a single monochromatic energy or spectra of 20 keV. The detector pixels had 100% detection efficiency.

We utilize measured scatter profiles, *I*, to inform the scattering properties of materials used in the simulation.

$$\begin{array}{c} I\left(q\right)=kF^{2}\left(q\right)S\left(q\right), \end{array}$$ (5)

where *k* is a constant, *k* = *I*_0_*nρ*^2^*V*^2^, that accounts for incident beam intensity, *I*_0_, number of unit molecules, *n*, density, *ρ* and volume of the particle, *V* . *F*(*q*) is the form factor of the scatter profile that considers the shape and size of a unit molecule, whereas *S*(*q*) is the structure factor that accounts for interference effects of unit molecules in close proximity. It is known that at sufficiently large *q*, the measured *F*^2^(*q*)*S*(*q*) asymptotically approaches the theoretical Independent Atomic Approximation (IAA) form factors, $F_{IAA}^{2}$, given by,

$$\begin{array}{c} F_{\text{IAA}}^{2}=\sum w_{Z}F_{Z}^{2}\left(q\right), \end{array}$$ (6)

where *w*_*Z*_ is the weight fraction of element *Z*, and *F*_*Z*_(*q*) is the coherent scatter form factor for element *Z*. [34–36] As a result, the absolute values of *F*^2^(*q*)*S*(*q*) could be estimated by re-normalizing the data to fit the $F_{IAA}^{2}\left(q\right)$ values in an interval of *q* ranging from 40 to 50 nm^−1^. We used the chemical composition for cortical bone and brain provided by the International Commission on Radiological Protection (ICRP) to calculate the independent atomic approximation form factors of these tissues. [37–39]

**Fig 4:**
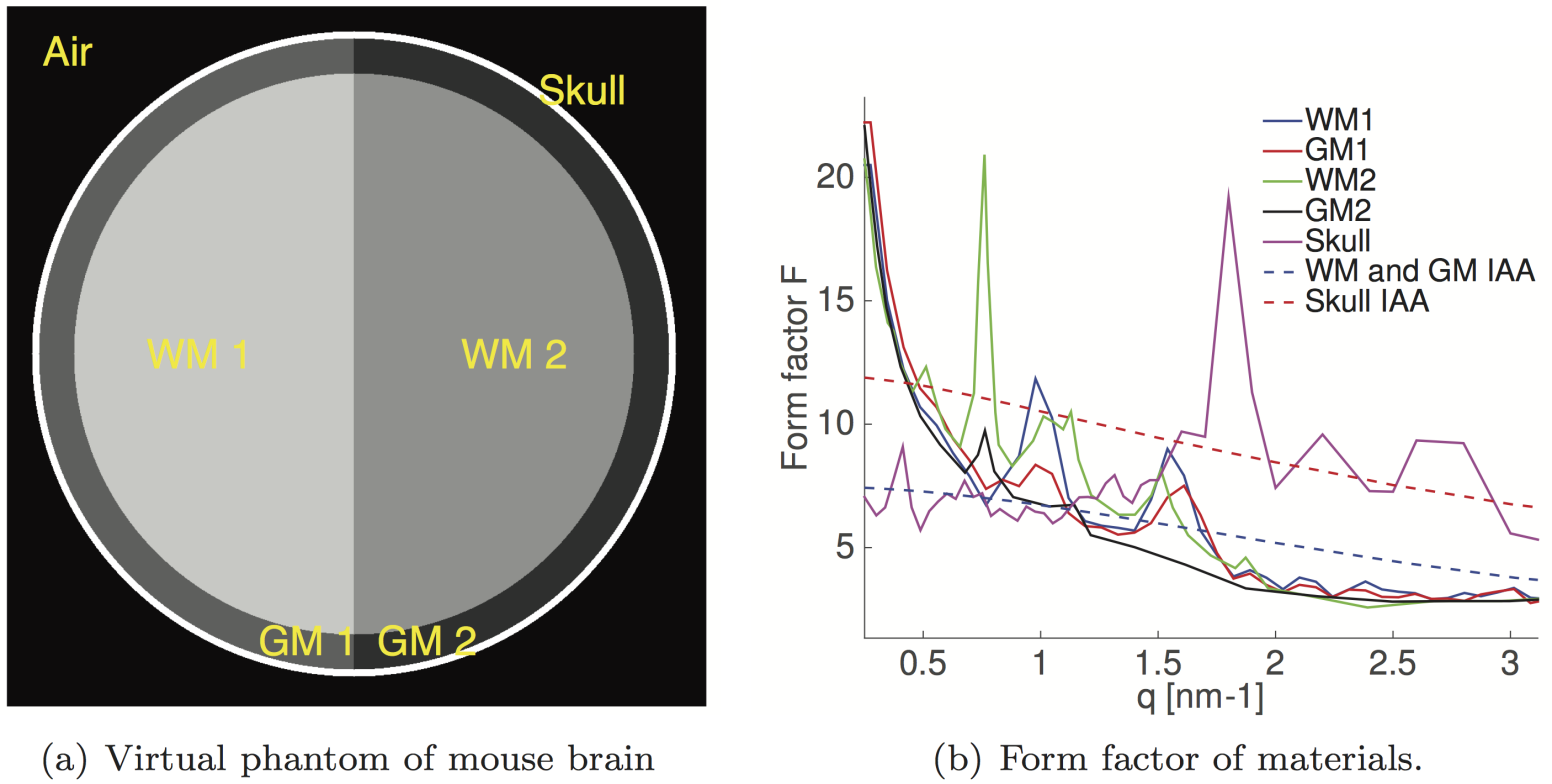
(a.) Simplified cylindrical model of a mouse head slice. (b.) Coherent scattering form factor for WM1, WM2, GM1, GM2 and skull materials. dotted line: form factors calculated with IAA. Solid line: form factors measured in this study.

In this study, we used our measured 1D scatter profiles *I*(*q*) of white matter (WM1), gray matter (GM1) and skull to improve scatter estimations at smaller angles where $F_{IAA}^{2}$ fails to approximate the S(q) effects. We also used the measured scatter profiles given by De Felici *et al.* [17] for WM2 and GM2 for comparison since differences in scatter profiles were observed for these materials. For WM1 and GM1 we used the same form factors of WM2 and GM2 respectively at wide angular range from 2.5–50 nm^−1^. For the skull we normalized our measured *F*(*q*) to those given by Tartari *et al.* [40] for bone in an interval of *q* ranging from 2.5 to 5 nm^−1^. Fig 4(a) shows the geometry of the cylindrical model of the mouse head. A comparison of *F*_*IAA*_(*q*) and measured $\sqrt{F^{2}\left(q\right)S\left(q\right)}$ are shown in Fig 4(b). The skull thickness was 0.2 mm, gray matter was 1 mm, and white matter was 8 mm in diameter. The density use for gray and white matter materials was 1.03 g/cm^3^ and the skull was 1.85 g/cm^3^. We simulated 100 translation points at 0.1 mm step sizes across this 1x1 cm^2^ region, and 360 projections with 1° angular steps. The x-ray energy was 20 keV monochromatic with a beam divergence of 0.08°. The sample-to-detector distance was 30 cm. The detector was 3 cm in radius and had 300 bins from the center to edge with a *q* range of 0–10 nm^−1^. For each translation and projection, we simulated 1×10^9^ histories totalling 3.6×10^13^ histories for the CT slice image with a total execution time of 13 hours running on 6 GeForce GTX Titan GPUs. To study the effect of the skull on signal quality and dose deposited to the brain, we repeated simulations replacing the skull material with WM1.

We calculated the signal-to-noise ratio (SNR) by taking the mean over the standard deviation of pixel values belonging to each material, *SNR* = *μ*_*mat*_/*σ*_*mat*_. This calculation could be achieved for each *q* intensity map, however, for the comparison of simulations with a skull in place versus skull replaced by gray matter, we selected a *q* angle with a prominent peak for both WM1 and WM2 at 1.03 nm^−1^.

MC-GPU calculates the energy deposited per gram and x-ray history (eV/g/history) and tabulates these values for each material simulated. We converted to mGy using the following equation, 1*Gy* = 6.24 × 10^15^ *eV*/*g*.

## Results

**Fig 5:**
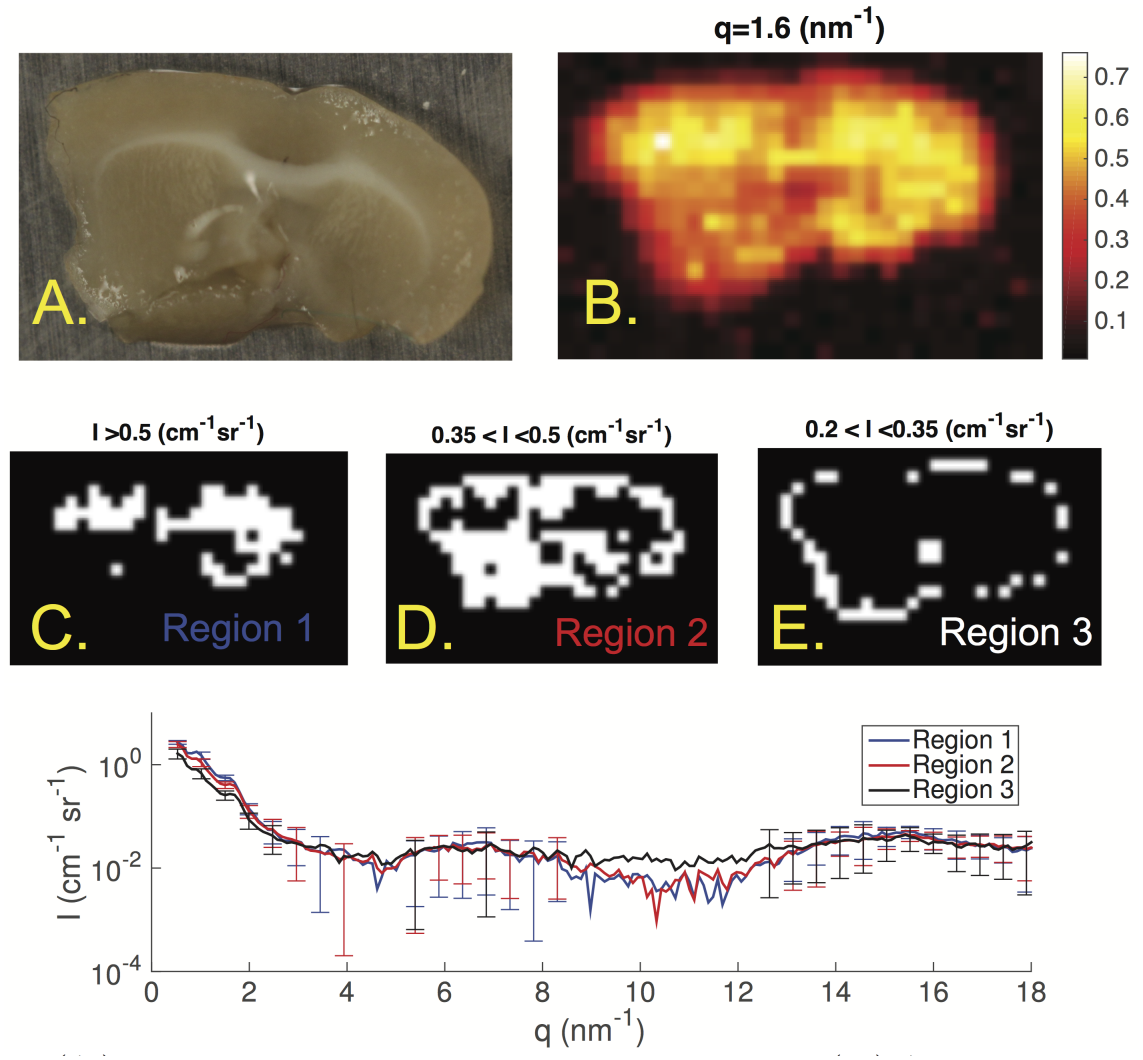
(A.) Photograph of a coronal slice in a mouse brain. (B.) An intensity map at *q* = 1.60 nm^−1^. (C.) First region where intensity was higher than 0.50 cm^−1^ sr^−1^ in B. (D.) Second region where the intensity was between 0.35 and 0.50 cm^−1^ sr^−1^ in B. (E.) Third region where the intensity was between 0.20 and 0.35 cm^−1^ sr^−1^ in B.(Bottom) Scatter profiles of the average of pixels in the three regions depicted in C,D and E. Error bars are ±*σ* of the pixels in each region. They are plotted every 10 points for clarity.

Fig 5 shows results from the first coronal slice of a mouse brain. We show the intensity map at 1.6 nm^−1^ where we observed a strong peak and three regions segmented by intensity from the intensity map. The average scatter profiles of pixels in the regions show a distinct peak at 0.96 and 1.60 nm^−1^ to varying intensities. The *q* resolution for these measurements were lower due to 8 × 8 binning. The higher binning allowed for higher signal intensity at wider angles where broad peaks occur and where high angular resolution is not needed. There is a broad peak at 6.75 and another at 15.4 nm^−1^.

**Fig 6:**
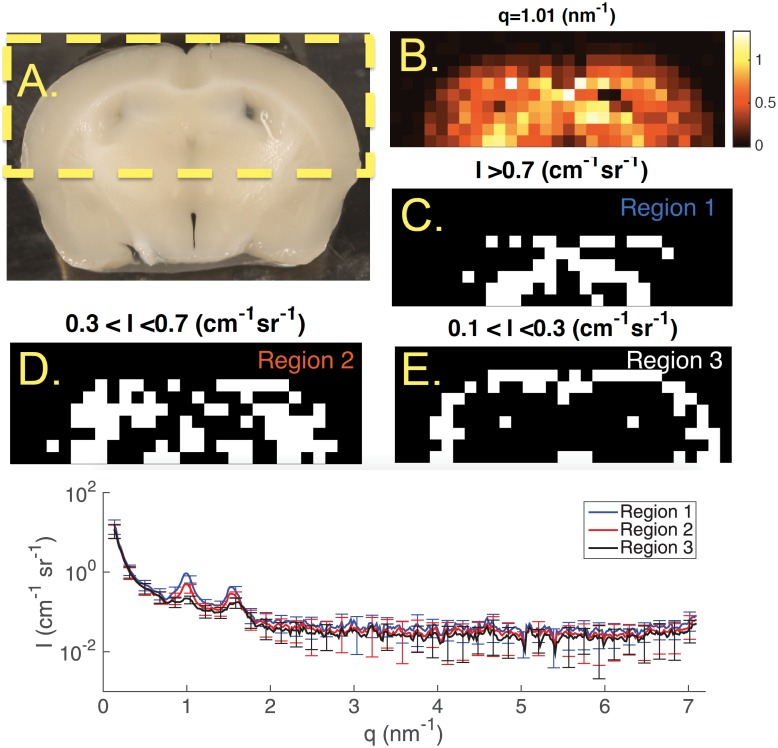
(A.) Photograph of a coronal slice in a second mouse brain. Dotted yellow line indicates region that was imaged. (B.) An intensity map at *q* = 1.01 nm^−1^ where the first peak appeared in the scatter profiles. (C.) First region where intensity was higher than 0.7 cm^−1^ sr^−1^ in B. (D.) Second region where the intensity was between 0.3 and 0.7 cm^−1^ sr^−1^ in B. (E.) Third region where the intensity was between 0.1 and 0.3 cm^−1^ sr^−1^ in B.(Bottom) Scatter profiles of the average of pixels in the three regions depicted in C,D and E. Error bars are ±*σ* of the pixels in each region. They are plotted every 10 points for clarity.

In Fig 6, we present pSAXS measurements of a slice from a second mouse brain at smaller *q* angles and range with 4 × 4 binning. The first peak existed for all positions at *q* = 1.01 nm^−1^, so we segmented three regions by the intensity map at that angle. The average scatter profiles of pixels in the regions shows there are two distinct peaks at 1.01 and 1.53 nm^−1^ also to varying intensities. All observed peaks in the scatter profiles, both distinct and subtle, are tabulated in Table 1.

**Table 1:** Compilation of the angular location of characteristic peaks.

	*q* (nm^−1^)			
Fig 5**, WAXS**	0.96	1.60	6.75	15.4
Fig 6**, SAXS**	1.01	1.53		

**Fig 7:**
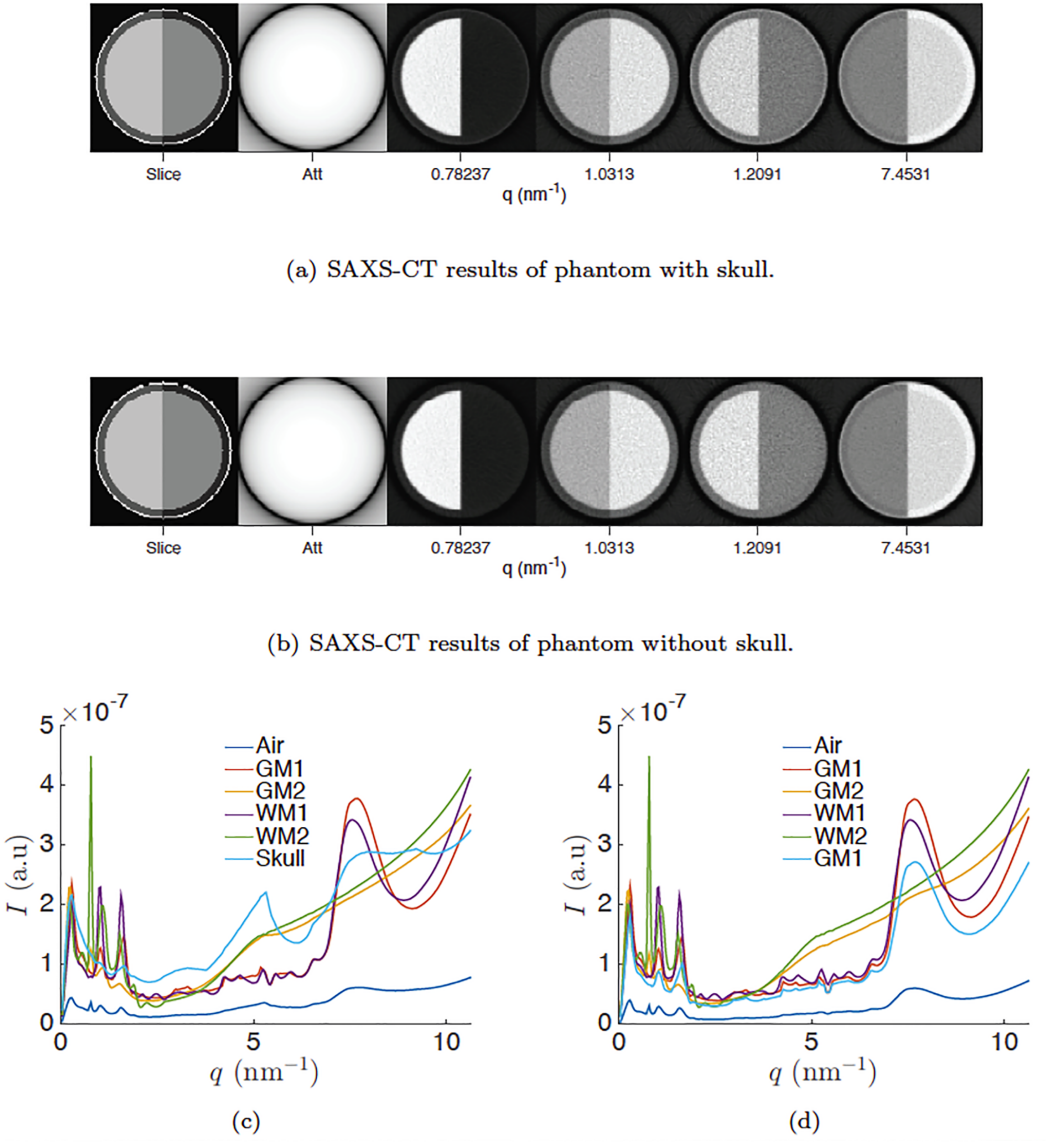
(a) Results from SAXS-CT simulations using MC-GPU. First image is a map of materials (same as Fig 1(a)). The second image is the attenuation image that a typical CT image would produce. The third and sixth images are intensity maps reconstructed from a specific *q* angle indicated below the image. (b) Results of SAXS-CT simulations with the skull replaced by GM2. (c-d) Using the material map in the first image, we averaged pixels belonging to a particular material and plotted cross sections of each material (c with skull, d without skull).

Fig 7 shows SAXS-CT simulations of a simplified mouse head constructed of cylinders. The outer layer is bone with a thickness of 0.2 mm. The next layer is gray matter with a thickness of 1 mm. The inner layer is white matter with a thickness of 8 mm. Also in Fig 7, we show material cross sections obtained from literature, whereas the right side uses material cross sections we measured. The CT images show at particular angles, the white matter material has more contrast with respect to other materials. By averaging the pixels belonging to each material type, we can reconstruct the scattering x-ray cross section of the materials. Because the skull is expected to be highly attenuating, we also simulated the same virtual phantom but with the skull voxels replaced with GM2 which is shown in the middle figure. All SAXS-CT images show presence of the skull. The simulation with skull resulted in an estimated total dose of 149.4 mGy whereas the simulation without skull resulted in an estimated 76.84 mGy deposited on the entire imaged object. In the simulation with skull, the dose delivered was 14.3 mGy to white matter, 14.8 mGy to gray matter, and 90.9 mGy to skull. Skull absorbed more x rays given that skull density was 1.85 g/cm^2^ and at the perimeter of the phantom whereas the brain density was 1.03 g/cm^2^ and in the center. In the simulation without skull, the dose delivered was 14.8 mGy to white matter and 15.6 to gray matter. The calculated SNR for each material is tabulated below in Table 2.

**Table 2:** SNR of each material for simulations with or without a skull. White matter (WM), Gray matter (GM).

	WM1	WM2	GM1	GM2
**with skull**	31.3 ± 0.97	30.4 ± 1.16	10.8	6.30
**without skull**	31.2 ± 0.30	30.3 ± 1.36	10.0	6.20

## Discussion

We imaged slices of mouse brain with an aim of characterizing the small-angle scattering cross section for various tissue types in the mouse brain and compare to results from others. A planar SAXS set-up allowed us to register different parts of the brain to SAXS intensity maps. Our planar SAXS measurements of three slices of normal wild-type mouse brain show regions in the slice of the brain with common characteristic cross section features, in particular, with the corpus callosum.

Some sources of error in our measurements are due to imperfect dark current subtraction since only one is obtained at the end. We mitigated some of the error by offsetting by a constant that is determined by averaging several points near the tail-end of the scatter profile that is supposed to be approximately 0 cm^−1^ sr^−1^. This reduced the standard deviation across all measurements at a few angles with approximately 0 cm^−1^ sr^−1^ in the scatter profile from 1.4201 to 0.1987 cm^−1^ sr^−1^ as shown in Fig 3 (right).

We scaled the measured intensity to absolute scale using a secondary glassy carbon standard. However, some error in absolute intensities are introduced with imperfect background subtraction. In a conventional SAXS measurement of bio-molecules in solution, it is advised to use the same quartz capillary sample holder for the signal measurement as well as the background measurement so the sample holder can be subtracted more accurately. In a planar SAXS imaging, it would be more robust to measure the same tissue sample holder without the tissue as a location-dependent background measurement, however this would double the measurement time and other issues may arise. The thin proprietary x-ray transparent plastic windows are not impervious to shifting when removing or positioning the brain tissue from the sample holder. In addition, the vacuum in the sample chamber imposes negative pressure on the sample holder windows which causes the window-to-window distances to be nonuniform across the scanned area. These issues should be considered in the design of a dedicated sample holder for tissue pSAXS measurements.

As a compromise for our background subtraction, we segmented regions of windows of the tissue sample holder which served as the background signal using intensity thresholds, averaged the scatter profile and used this as a surrogate background for background subtraction.

We were able to detect prominent peaks that exist in white matter and more so in the corpus callosum structure which we suspect could be due to the higher density of nerve fibers and myelin sheaths surrounding the nerve axons. Myelin has been reported to be a strong small-angle scatterer and the subject of many neurological SAXS studies. The prominent peak was at 0.92 nm^−1^ which is slightly higher than results from Lazarev *et al.* and similar to 0.76 nm^−1^ from De Felici *et al.* and 0.75 nm^−1^ from Jensen *et al*. The differences can be explained by the *q* resolution and uncertainty of our system, [41] but also by differences in myelin and neuron structure in different species of animals as previous reports were on human and rat brain. Finally, the drying of the brain before measurements may shrink the periodic packaging of the myelin layers thereby overestimating peak location.

Other peaks reported by others were not detected in our measurements. This is probably because of a combination of low scatter signal intensities and peak broadening due to our *q* resolution. Higher quality measurements can always be performed at a synchrotron source where pencil beam sizes can go down to 10 *μ*m^2^, there is flexibility in energy of x-rays, and flux of the beam is between 10^11^ to 10^13^ photons/s, where SAXS-CT measurements were demonstrated before. [14] However, we have demonstrated detection of the strongest myelin peak within the corpus callosum structure with a laboratory source.

The SAXS-CT simulations showed that the approximate dose to the mouse head for a single CT slice imaged was approximately 149.4 mGy. Improvements can be made by using higher energy x rays. Further simulation studies that sweep the monochromatic energy used would be required to ascertain which x-ray energy produces the most scatter signal in a small-animal or human head while minimizing radiation dose. However using 20 keV x rays, we were successfully able to reconstruct the original cross sections of each pixel location in the CT slice albeit a high dose.

## Conclusion

We report measured small-angle x-ray scattering (SAXS) of mouse brain slices in a planar imaging mode to characterize cross sections of various tissue types within the brain. We then compare results from mouse brain to previous SAXS measurements of brains from other species and aims to generalize commonalities in cross section peaks attributed to myelin, which is the strongest scatterer within the brain. Our findings demonstrate SAXS-CT with simulations using a Monte Carlo x-ray transport simulator (MC-GPU) of a simplified mouse head model and report estimated SNR and radiation dose levels.
